# The importance of histological patterns on PD-L1 staining heterogeneity: Should we use pattern-based approach for selecting tumor samples for PD-L1 testing in lung adenocarcinomas?

**DOI:** 10.3906/sag-2004-61

**Published:** 2021-02-26

**Authors:** Pınar BULUTAY, Pınar FIRAT, Handan ZEREN, Suat ERUS, Serhan TANJU, Şükrü DİLEGE

**Affiliations:** 1 Department of Pathology, Medical Faculty, Koç University, İstanbul Turkey; 2 Department of Pathology, Medical Faculty, Acıbadem University, İstanbul Turkey; 3 Department of Thoracic Surgery, Medical Faculty, Koç University, İstanbul Turkey

**Keywords:** PD-L1, lung cancer, immunotherapy, lung adenocarcinoma

## Abstract

**Background/aim:**

Programmed death ligand-1 (PD-L1) is a predictive marker for immunotherapeutic agents. However, heterogeneous staining of PD-L1 can cause false-negative results. The aim of this study is to evaluate the importance of histological patterns on PD-L1 staining heterogeneity in lung adenocarcinomas (LAC). **Materials and methods** **:** PD-L1 immunohistochemistry (IHC) stain was performed to two different tissue cores of 128 LAC cases, and cut-off values are given for grouping the cases according to the percentage of staining (1%-10%, 11%-49%, 50%-100%). Staining rates between cores were compared and analyzed by their histological patterns. Also, the relation of the PD-L1 expression with the clinicopathological characteristics of the cases was analyzed.

**Results:**

Overall, PD-L1 expression was observed in 53 of 128 cases (41.4%, 1% cut-off), 23.5% of them were positive at 10% cut-off and 14.1% at 50% cut-off. PD-L1 expression was significantly related to the high grade micropapillary and solid patterns of adenocarcinomas (p:0.01). Staining cut-offs were mostly similar between cores (43/50, 86%) (k:0.843). However, 14% of them were positive only in one core (7 of 50). This false negativity was mostly related to the histological patterns.

**Conclusion:**

Our data reveal the heterogeneous staining of PD-L1 expression, also micropapillary and solid patterns show higher rates of PDL expression. Therewithal, these findings also highlight the importance of taking into consideration of histological patterns, when choosing a paraffin block for the PDL1.

## 1. Introduction 

Lung cancer is the leading cause of cancer related mortality worldwide and one of the most highly mutated ones among solid tumors. Many lung cancer patients have a high mutational burden [1]. Nonsmall cell lung cancer (NSCLC) accounts for the majority of lung cancer cases (80%-85%) [2,3]. Most patients have locally advanced or metastatic disease on initial presentation. In the past, the treatment options for advanced or metastatic disease were typically confined to chemotherapy or radiation therapy, but the advent of targeted therapies as EGFR (epidermal growth factor receptor) and ALK (anaplastic lymphoma kinase) inhibitors have led to improved outcome in some patients who harbor driver oncogenes, especially in lung adenocarcinomas [4,5].

Recently immunotherapy represented a new and highly promising therapeutic option for metastatic NSCLCs on first and second-line therapy. Several approved immunotherapeutic drugs, such as pembrolizumab, avelumab, and nivolumab are being used on the ‘programmed cell death-ligand 1 (PD-L1) positive (≥ 1%) cases. Guidelines for NSCLC treatment emphasizes the importance of PD-L1 expression levels for optimal use of antiPD1/PD-L1 therapies with or without chemotherapeutic agents. Single-agent pembrolizumab can be used in NSCLC patients with PD-L1 expression higher than 50% in tumor cells [6]. 

PD-L1 is a transmembrane protein and normally expressed on the antigen-presenting cells and also some tumor cells [7–9]. It is one of the most important immune-inhibitory checkpoints, and it can stop or limit the development of the T-cell response through binding to its inhibitory receptor, programmed death-1 (PD1). PD1 is an inhibitory receptor located on the surface of activated T, B, and natural killer cells [10,11]. An interaction between the PD1 receptor and PD-L1 leads to inhibition of primary T-cell proliferation response and cytolytic activity against the tumor antigens and protects the tumor cells from the antitumor immune response. At this point, the immune checkpoint inhibitors, against either PD1 or PD-L1 and reactivate the immune system and tumor cells become visible again [12]. 

PD-L1 immunohistochemistry (IHC) is an established method for testing intratumoral PD-L1 expression in daily practice [13]. However, it has some difficulties. The biggest obstacle is PD-L1 can show heterogeneous expression, so IHC results can lead to false negative results, especially on small biopsy specimens [14–20].

In this study, we retrospectively analyzed the PD-L1 expression of resected lung adenocarcinomas and analyzed the importance of histological patterns on heterogeneous expression with the microarray technique. 

## 2. Materials and methods

This study comprises 128 lung adenocarcinoma cases that had undergone surgery at the Koç University Hospital and the American Hospital (Turkey) between 2011 and 2017. Clinical and pathological data were recorded using electronic medical files and pathology reports. The hematoxylin and eosin (H&E) stained slides were retrieved from the pathology archives and reviewed by two expert pulmonary pathologists (PB and PF). All samples were reclassified and restaged according to 2015 WHO classification and TNM staging (8th edition) for lung carcinomas [21]. 

Two separate tumor areas were selected in a 4 mm diameter on H&E-stained slides and removed from the corresponding areas of paraffin-blocks for tissue microarray (TMA) construction. We selected one core from the dominant pattern and the other from the high-grade pattern, if present. Nine of the cases were studied only on one core because of the tumor size. Totally 16 new TMA paraffin blocks were constructed. Two unstained sections were taken from the paraffin blocks, one of them was stained with H&E and the other stained with PD-L1 IHC. Histological patterns were evaluated of each tissue cores independently. The lepidic, papillary, and acinar patterns were recorded as low/intermediate grade, and the solid, micropapillary patterns, and mucinous adenocarcinomas were recorded as high grade [22,23].

### 2.1. Immunohistochemistry

Immunohistochemistry on TMA sections was carried out with an automated stainer (Ventana Benchmark (Tucson, AZ) using antiPD-L1 (SP142) with optiview detection kit, obtained from Roche (Arizona, USA). Tissue samples were considered adequate for evaluation if the tissue samples were had more than 100 tumor cells and classified as positive if the expression was seen in at least 1% of tumor cells with complete circumferential or partial linear membranous staining at any intensity [24].

### 2.2. PD-L1 scoring

PD-L1 evaluation was performed blindly and independently for each core (PB). PD-L1 expression was scored semiquantitatively according to the percent of PD-L1 positive tumor cells. The staining scores were given separately for each core according to the percentage of staining (1%–10%, 11-49%, 50%–100%). The mean PD-L1 score of two cores was recorded as a PD-L1 score of the case. Tonsil tissue was used as external control, whereas macrophages were used as an internal control. 

### 2.3. Statistical analysis

Statistical analysis was performed by the SPSS software program version 24.0. (IBM Corp., Armonk, NY, USA). The relation of PD-L1 expression with clinicopathological parameters and histological patterns was investigated using Pearson’s χ2 or Fisher’s exact test. Cohen’s kappa coefficient was used to compare the agreement between two cores. Overall survival (OS) rates were calculated via the Kaplan-Meier method. Independent samples T-test was used to assess the relationship between age, tumor size, and PD-L1 expression. P values < 0.05 were considered as statistically significant.

## 3. Results

**Figure 1:**
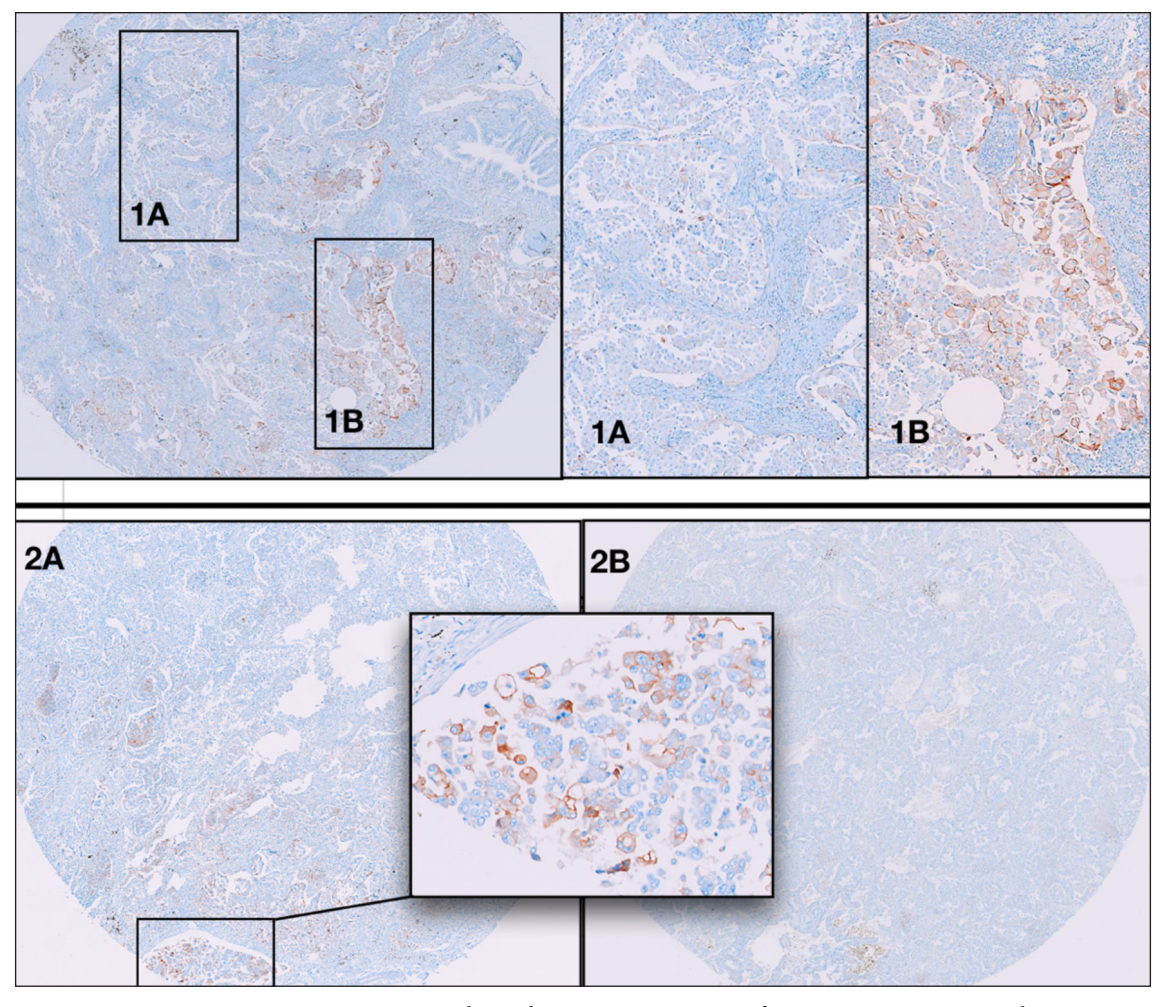
Heterogenous staining pattern samples with PD-L1. 1A: Negative for PD-L1. 1B: Positive with PD-L1. 2A: First core with 5% staining with PD-L1 in micropapillary pattern. 2B: Second core negative for PD-L1 in acinar pattern.

**Figure 2:**
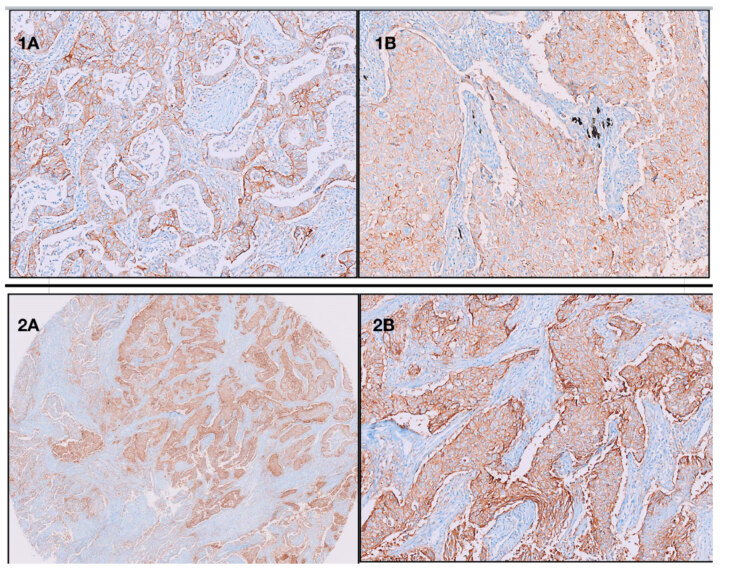
1A: PD-L1 staining in acinar pattern. 1B: PD-L1 staining in solid pattern. 2A: 100% positive staining of PDL1 in solid pattern (≥ 50% cut-off). 2B: Higher magnification (×20).

PD-L1 expression was identified in 41.4% (53/128) of the cases at ≥ 1% cut-off. The positivity rate was 23.5% at > 10%, and 14.1% at ≥ 50% cut-off values. PD-L1 expression was significantly more common in high grade (solid and/or micropapillary) predominant tumors at all cut-off values (≥ 1 (P = 0.14), > 10 (P = 0.03), ≥ 50 (P = 0.01)). We found that there was a greater frequency of tumor size with PD-L1 expression (P = 0.048). However, no clinicopathologic variable correlated with age, gender, lymph node metastasis, pleural invasion, stage, lymphovascular invasion, spread through air spaces (STAS) (P > 0.05), and overall survival (Table 1). As mentioned in the materials-methods section, only one tissue sample/one core was taken from 9 cases (7%). Three of them were positive with PD-L1 (5.6%, 3/53). Since the purpose of our study was to investigate the difference in staining rates between two cores, we excluded these 3 cases from the study. Forty-three of the positive cases (86%, 43/50) was sharing PD-L1 expression on both cores (cut-off value ≥1%). In 30 cases, the PD-L1 expression rate in both cores was at the same cut-off value (69%, 30/42). Thirteen cases were expressing PD-L1 at different cut-off values though (31%, 13/42) (Figure 1-1a, 1b). However, 7 cases were positive in only one core (14%, 7/50) (Figure 1-2a, 2b). In many of these, the positive core was at a 1%-10% cut-off value (5/7). Others were at a cut-off value of 11%-50% (2/7). Table 2 provides further details and cut-offs of the PD-L1 expression on both cores for each case. 

**Table 1:** Relationship of PD-L1 expression with the clinicopathological features of the cases

Variables	PD-L1 ≥1% N (%)			PD-L1 >10% N (%)			PD-L1 ≥50% N (%)		
	Positive	Negative	P	Positive	Negative	P	Positive	Negative	P
Sex									
Female	26 (46.5%)	30 (53.5%)	0.309	13 (23.3%)	43 (76.7%)	0.894	9 (16.1%)	47 (83.9%)	0.564
Male	27 (37.5%)	45 (62.5%)		16 (21.7%)	58 (78.3%)		9 (12.4%)	63 (87.5%)	
T Stage									
T1	35 (38.1%)	52 (61.9%)	0.694	15 (17.3%)	72 (82.7%)	0.16	9 (10.4%)	78 (89.6%)	0.78
>T1	18 (55%)	23 (45%)		14 (34.2%)	27 (65.8%)		9 (22%)	32 (78%)	
N Stage									
N0	32 (36.8%)	55 (63.2%)	0.122	18 (20.7%)	69 (79.3%)	0.439	12 (13.8%)	75 (86.2%)	0.898
N1 + N2	21 (51.3%)	20 (48.7%)		11 (26.9%)	30 (73.1%)		6 (14.7%)	35 (85.3%)	
Stage									
1	36 (40%)	54 (60%)	0.619	20 (22.3%)	70 (77.7%)	0.857	11 (12.3%)	79 (87.7%)	0.357
>1	17 (45%)	21 (55%)		9 (23.7%)	29 (76.3%)		7 (18.5%)	31 (81.5%)	
Pleural invasion									
Absent	31 (42%)	43 (58%)	0.896	21 (28.4%)	53 (71.6%)	0.07	12 (66.7%)	6 (33.3%)	0.412
Present	22 (40.8%)	32 (59.2%)		8 (14.9%)	46 (85.1%)		62 (56.4%)	48 (43.6%)	
Venous and lymphatic invasion									
Absent	31 (46.5%)	38 (53.5%)	0.382	18 (27.7%)	48 (72.7%)	0.316	11 (61.2%)	7 (38.8%)	0.508
Present	22 (37.3%)	37 (62.7%)		11 (17.8%)	51 (82.2%)		58 (52.7%)	52 (47.3%)	
STAS									
Absent	23 (46.9%)	25 (53.1%)	0.247	14 (29.2%)	34 (70.8%)	0.173	8 (16.7%)	40 (83.3%)	0.512
Present	30 (37.7%)	50 (62.5%)		15 (18.8%)	65 (81.2%)		10 (12.5%)	70 (87.5%)	
Dominant pattern									
Low grade	31 (34.5%)	59 (65.5%)	0.014	15 (16.5%)	76 (83.5%)	0.03	8 (6.9%)	82 (93.1%)	0.01
High grade	22 (41.6%)	31 (58.4%)		14 (37.9%)	23 (62.1%)		10 (26.4%)	28 (73.6%)	
Age	Median age (range): 63.2 (32-86))				P = 0.98				
Tumor size	Median size (Range): 2.61 (1.4-8 cm)				P = 0.048				
Overall survival(median) (60 moths)	PDL1 (+): 64 moths. PDL1 (-): 54 moths.				P = 0.59				

**Table 2:** Histologic patterns and staining rates of positive cases with PD-L1.

Case no	1st core pattern	2nd core pattern	1st core PD-L1 score	2nd core PD-L1 score
1	Solid	Solid	3%	3%
2	Micropapillary	Acinar	5%	0%
3	Micropapillary	Solid	40%	40%
4	Acinar	Acinar	5%	2%
5	Acinar	Acinar	2%	2%
6	Micropapillary	Micropapillary	10%	10%
7	Solid	Acinar	30%	0%
8	Solid	Solid	30%	10%
9	Solid	*	30%	*
10	Solid	Solid	5%	5%
11	Solid	Solid	2%	2%
12	Solid	Acinar	100%	30%
13	Papillary	Papillary	3%	3%
14	Micropapillary	Acinar	3%	0%
15	Solid	*	3%	*
16	Micropapillary	Papillary	2%	2%
17	Papillary	Papillary	2%	5%
18	Solid	Acinar	10%	5%
19	Micropapillary	Micropapillary	3%	1%
20	Solid	Micropapillary	5%	30%
21	Micropapillary	Acinar	20%	0%
23	Solid	Solid	70%	80%
23	Acinar	Acinar	7%	2%
24	Solid	Solid	1%	0%
25	Solid	Acinar	80%	20%
26	Solid	Papillary	5%	0%
27	Solid	Solid	0%	5%
28	Papillary	*	2%	*
29	Papillary	Solid	5%	10%
30	Solid	Papillary	100%	100%
31	Acinar	Acinar	100%	100%
32	Solid	Solid	60%	50%
33	Solid	Solid	50%	60%
34	Solid	Acinar	100%	80%
35	Solid	Solid	5%	5%
36	Solid	Micropapillary	100%	50%
37	Micropapillary	Solid	60%	30%
38	Solid	Solid	80%	80%
39	Papillary	Papillary	2%	2%
40	Acinar	Micropapillary	30%	40%
41	Acinar	Acinar	80%	80%
42	Papillary	Papillary	80%	80%
43	Solid	Solid	5%	5%
44	Solid	Solid	5%	5%
45	Acinar	Acinar	3%	0
46	Solid	Solid	30%	50%
47	Solid	Solid	50%	50%
48	Acinar	Acinar	5%	20%
49	Solid	Solid	30%	60%
50	Solid	Papillary	5%	1%
51	Solid	Solid	100%	100%
52	Solid	Solid	10%	5%
53	Solid	Solid	60%	70%

*Single core studied cases.

### 3.1. PD-L1 staining and histological patterns 

A total of 247 tissue cores were obtained from 128 cases. The distribution of histological patterns in these cores was as follows: 99 acinar (Figure 2-1a), 88 solid (Figure 2-1b, 2-2a, 2-2b), 32 papillary, 21 micropapillary, 5 lepidic pattern, and 2 mucinous adenocarcinomas. Among these, the most frequent positive PD-L1 rate was included micropapillary (66.7%, 14/21) and solid patterns (59.2%, 51/88). Subtype groups with the least frequent PD-L1 expression included papillary (40.6%, 14/21) and acinar (19.4%, 18/99) subtypes. PD-L1 positivity was not seen in the lepidic pattern (0%, 0/5) and mucinous adenocarcinomas (0%, 0/2). We found that there was a greater frequency of PD-L1 expression with high-grade histological patterns (solid and micropapillary) compared with the low-intermediate grade histological patterns (lepidic, acinar, papillary) (P = 0.001) (Table 3). 

**Table 3:** PD-L1 staining rates in all cores (119 [double cores] ×2+9 [single cores]) = 247) between low- and high-grade histologic patterns.

	Negative	> 1%	> 10%	≥ 50%	Total	P
Lepidic + acinar + papillary	105 (70%)	19 (41.3%)	4 (20%)	8 (25.8%)	136 (54.9%)	0.001
Micropapillary + solid + mucinous adenocarcinoma	45 (30%)	27 (58.7%)	16 (80%)	23 (74.2%)	111 (45.1%)	
Total	150 (100%)	46 (100%)	20 (100%)	31 (100%)	247 (100%)	

Although in a few cases negativity is observed in one core, according to Cohen’s test overall agreement between two cores were ‘strong’ for all cut-off values (respectively 84%, 84%, and 81%) [25] (k ≥ 1%: 0.843, k > 10%: 0.848, k ≥ 50%: 0.815) (Table 4). 

**Table 4:** Concordance between cores at different cut-offs.

1% cut-off					
	2nd core				
1st core		Negative	≥ %1	Total	κ 0.843
	Negative	67 (98.5%)	1 (1.5%)	68 (100%)	
	≥%1	8 (16%)	43 (84%)	51 (100%)	
	Total	75 (63%)	44 (37%)	119 (100%)	
10% cut-off					
	2nd core				
1st core		Negative	≥ %1	Total	κ 0.848
	Negative	91 (97%)	3 (3%)	94 (100%)	
	≥%1	3 (12%)	22 (88%)	25 (100%)	
	Total	94 (79%)	25 (21%)	119 (100%)	
50% cut-off					
	2nd core				
1st core		Negative	≥ %1	Total	κ0.815
	Negative	101 (96%)	2 (4%)	105 (100%)	
	≥%1	3 (19%)	13 (81%)	16 (100%)	
	Total	104 (87.3%)	15 (12.7%)	119 (100%)	

## 4. Discussion

PD1/PD-L1 receptor-ligand binding is a dominant immune checkpoint pathway, which is known to contribute to tumor immune evasion in several cancer types particularly NSCLC [26,27]. Thus, immune checkpoint inhibitors represent an important breakthrough in cancer treatment and have demonstrated to be highly effective in many tumor types [28,29]. Recently, the U.S. Food and Drug Administration (FDA) approved an antiPD1 drug, as a single chemotherapeutic agent as first-line therapy in patients with tumors expressing PD-L1 in at least 50% of neoplastic cells and second-line therapy with or without chemotherapy combination in patients with more than 1% PD-L1 expression [6,30]. IHC analysis of PD-L1 expression is being used to identify patients who may benefit from PD1/PD-L1 inhibitors [13]. However, heterogeneous staining characteristics of PD-L1 may give rise to false-negativity especially in small biopsy samples [31]. In this study, we aimed to provide a more accurate histological pattern-based approach to intratumoral heterogeneity of PD-L1 expression in lung adenocarcinomas. 

As is well-known, most lung adenocarcinomas exhibit mixed histological patterns. In 2011, the International Association for the Study of Lung Cancer, American Thoracic Society, and European Respiratory Society proposed a new histological classification for lung adenocarcinomas [32]. This classification recognizes the major histological patterns (lepidic, acinar, papillary, solid, and micropapillary) and variants (mucinous, colloid, enteric, and fetal). Lung adenocarcinomas are labeled according to the predominant histological pattern after this classification [21]. Among these histological patterns, solid and micropapillary patterns have a worse prognosis than lepidic, acinar, and papillary patterns [33,34]. 

The present immunohistochemical study examined 128 resected lung adenocarcinomas in order to evaluate the heterogeneous expression of PD-L1 between different parts of the tumors and correlations with histological patterns and clinicopathological parameters and potential prognostic impact of PD-L1 expression. Our overall PD-L1 expression rate was 43.4% at >1% cut-off, and 14.1% at > 50% cut-off values. Similar results have been obtained in some other studies [20,35]. Moreover, in some series showed a higher percentage of PD-L1 positive cases than we have found [13,14]. This may due to macroscopic sampling conditions due to delayed/impaired fixation since all cases are resection specimens or may be attributable to the retrospective nature of this study. Gagne et al. demonstrated that specimens containing fewer than 100 tumor cells or older than 3 years may lead to an underestimation of PD-L1 status [36]. At this point, almost half of our cases were older than 3 years. However, all specimens were prepared in the same laboratory with the same standards, and they were reevaluated by the same pulmonary pathologists. Thus, the results of our study, especially the comparison of the cores, can be considered relatively robust. Another relevant finding with the poor prognosis was that the majority of cases with PD-L1 expression results had a larger tumor size. PD-L1 expressing tumors can reach larger diameters than the others. Similar results have been obtained regarding tumor size and PD-L1 expression in lung adenocarcinomas [37]. In addition, the same study showed that PD-L1 expression was also associated with male gender, smoke, lymph node metastasis, EGFR wild-type status, KRAS mutations, and overall survival [37]. However, we did not find any significant relationship between age, sex, lymph node metastasis, pleural invasion, lymphovascular invasion, STAS, and PD-L1 expression. Also, no significant correlation was found with overall survival; the follow-up periods of the cases were rather short (our mean follow-up time: 62 months). Therefore, a longer follow-up period may be necessary for a more accurate evaluation.

Another interesting finding was the significant correlation among dominant histological patterns and PD-L1 expression. That is the PD-L1 expression rate increases as the tumor differentiation decreases. Song et al. correlated the PD-L1 expression and clinicopathologic features in 404 lung adenocarcinoma patients, and they showed the relation between solid predominant subtype and PD-L1 staining [38]. Similar results have been found in two different studies. In these studies, PD-L1 expression in tumor cells has a correlation with a high histological grade and solid subtype, likewise our results [39,40]. Furthermore, another aim of this study was to analyze the staining differences between different cores. Inside of the positive cases, 14% of them were one core negative. Therefore, taking two different samples from different parts of tumor samples has increased PD-L1 positivity rate in our series. Munari et al. built tissue microarrays with 5 cores per case from 268 cases and compared PD-L1 staining results in the cores with the results obtained by using whole tumor sections [41]. According to their study, 3 or 4 cores are necessary to reach the lowest number of false-negative cases at both cut-offs as 1% and 50%. However, the size of the cores in their study was 1 mm, whereas those included in the present study were 4 mm. Furthermore, most of both core positive cases (69%, 29/42) were positive in the same cut-off values. Nevertheless, 31% (13/42) of them were positive at different cut-offs. This finding highlights the heterogeneity of PD-L1 staining also in our series. Haragan et al. quantified the heterogeneity by comparing different samples from the same tumor at different scales/magnifications; they found intra-tumoral heterogeneity rate decreases if the sections are examined at high power as 78% at small-scale and 46% at large-scale [42]. The primary objective of many studies in the literature is to minimize the number of false-negative cases to ensure that all eligible patients benefit from immunotherapy. In this context, the second question of our study was whether we could relate this heterogeneous staining with histological patterns of lung adenocarcinomas and predict the positivity rate of PD-L1 expression according to the histological patterns of the tumor. Our data indicate histological patterns of lung adenocarcinoma is related to the PD-L1 expression. In our one core unstained cases, 75% of unstained cores had low/intermediate grade (acinar/papillary) histologic patterns (62.5% acinar, 25% solid, 12.5% papillary patterns). Likewise, the PD-L1 expression rate was significantly higher in solid and micropapillary patterns as compared with acinar, lepidic, and papillary patterns. Two recent studies also showed similar findings; according to their results, PD-L1 positivity was seen mostly in solid/micropapillary patterns in lung adenocarcinoma cases [40,43]. These findings were consistent with another study, which reported an association between PD-L1 expression and histological patterns in pulmonary adenocarcinomas [44]. 

Finally, PD-L1 expression rates between two cores have shown a strong agreement according to the Cohen test (k = 0,843). Although, the remaining 25% of cases had a noncompliance though. If we look at the staining rates of one core unstained cases, none of them was staining higher than 50%. These results leading to results of a higher number of biopsies may increase the number of positive cases, especially in low expression rates, in other words, obtaining additional cores may help to better assess the PD-L1 status. [45]. Haragan et al. say that increasing quantities of tissue for assessment will clearly improve the accuracy, but in fact, even a whole tissue section might still not be representative of the entire tumor [42]. 

This study has some limitations. First, the PD-L1 expression of the cores was not compared with the whole tumor sections. However, as we obtained two 4-mm tissue cores from each tumor, the samples can be considered as clinically representative biopsies. Second, this is a retrospective study, and preanalytical issues or long archival period may affect the PD-L1 expression status [16]. However, an updated analysis of the Keynote-010 trial study [46], the authors compared the PD-L1 expression status in archival versus newly collected tumor samples. According to their results, the distributions of PD-L1 expression levels (≥ 1% and ≥ 50%) were similar among both archival (60% and 45%, respectively) and newly collected (55% and 45%) tumor samples. 

## 5. Conclusion 

This study reflects the correlation between histological patterns and staining heterogeneity of PD-L1 expression in lung adenocarcinomas. This heterogeneous staining may lead to false negativity in some cases especially in small biopsy samples. The tumors showing solid or micropapillary patterns had more frequent PD-L1 expression when compared to acinar, papillary, and lepidic patterns. According to our results, obtaining more samples from tumors will increase the accuracy of PD-L1 status and solid and/or micropapillary areas may be favored for PD-L1 testing if the tumor has. 
